# A novel technique for treating simple transverse patellar fractures using cannulated screws: a cadaveric and clinical study

**DOI:** 10.1186/s13018-023-04309-z

**Published:** 2023-11-06

**Authors:** Feng Han, Zhenjia Zhong, Ming Zhou, Qi Chen, Yinan Liu, Yongjun Rui, Fengfeng Li

**Affiliations:** 1 Department of Hand Microsurgery, First Affiliated Hospital of Dalian Medical University, Dalian, China; 2 Department of Orthopedics, The Second Affiliated Hospital of Guangzhou Medical University, Guangzhou, China; 3 Department of Orthopedics, Wuxi 9th People’s Hospital Affiliated to Soochow University, Wuxi, China; 4 First Affiliated Hospital of Dalian Medical University, Dalian, China; 5 Guangzhou Medical University, Guangzhou, China; 6 Department of Orthopedic Surgery, Nanjing Drum Tower Hospital, Affiliated Hospital of Medical School, Nanjing University, Nanjing, China

**Keywords:** Transverse patellar fracture, Cannulated screw, Minimally invasive, Closed reduction and fracture fixation

## Abstract

**Background:**

Tension band wiring (TBW) has conventionally been used for the open reduction and internal fixation of the patella. However, it suffers from distinct disadvantages such as large incision, implant irritation, and need for subsequent implant removal. Here, we propose a novel technique using closed reduction and percutaneous fixation with three cannulated screws (TCS), which may be an alternative to this established conventional technique. Although some researchers have proposed alternative methods including closed reduction and cannulated screw fixation, with or without additional wires through the screws, and arthroscopic-assisted reduction and fixation, there are few studies that focus on the biomechanical stability of percutaneous fixation using only cannulated screws. Thus, the purpose of this study was to evaluate TCS versus TBW for simple transverse patellar fractures in cadaveric and patients' level, aiming to determine whether TCS show superiority over TBW in terms of biomechanical stability in a cadaveric study with benign clinical feasibility and outcomes in patients.

**Methods:**

We conducted a cadaveric study with 15 knee specimens that had simple transverse patellar fractures. We used two fixation techniques: TBW (group A, *n* = 6) and TCS (group B, *n* = 9). We applied sinusoidal forces (25 N–125 N) at 1/5 Hz and 90° knee flexion to simulate knee movement. We compared the displacements at the fracture site between the two groups. We also used the same technique in a total of 23 patients and followed up them for at least 1 year.

**Results:**

TCS demonstrated favourable biomechanical stability in the cadaveric study. The technique also performed excellently in terms of postoperative pain, knee function recovery, and complication rates during the follow-up period.

**Conclusions:**

The technique provides a surgical treatment option with small incisions, minimal soft tissue irritation, and possibly lower removal rate of bothersome material.

## Introduction

Among various patella fractures, the most common type is the transverse fracture, accounting for approximately 50–80% of all patellar fracture types [1, 2]. Currently, the most conventional surgical technique after transverse patella fractures is TBW [3, 4]. A tension band construct is a surgical technique that applies a wire or a suture to the tension side of a bone fracture, converting the distractive forces produced by the extensor mechanism and knee flexion into compressive forces across the fracture site. This helps to achieve stable fixation and promote healing. However, this technique has some disadvantages, such as requiring extensive soft tissue dissection, which may compromise the blood supply and viability of skin flaps. Moreover, it may cause irritation or infection of the hardware, leading to removal in up to 60% of patients [5–7]. In addition, TBW technique necessitates a long skin incision and a large exposure of the fracture and joint surfaces, resulting in adhesion formation, prolonged disability, and poor cosmetic outcome.

Alternative methods have been proposed to treat simple transverse patellar fractures to avoid the drawbacks of TBW. These methods include closed reduction and cannulated screw fixation, with or without additional wires through the screws, and arthroscopic-assisted reduction and fixation [8–14]. These techniques are less invasive than TBW and preserve the blood supply to the patella, leading to faster recovery and fewer complications [8, 10, 13, 15]. Besides a meta-analysis performed by Lo et al. [16] compares the outcomes of minimally invasive percutaneous fixation and open reduction internal fixation for patella fractures and concludes that the former is more favourable than the latter in terms of pain, range of motion, joint functionality, complications, and implant removal. Lin et al. [17] conducted a randomized controlled trial (RCT) to compare CRPF and open reduction and internal fixation (ORIF) for simple transverse fractures (< 8 mm gap) and prove the former's superiority in terms of clinical outcomes. However, the biomechanical stability of CRPF using only cannulated screws has not been well studied compared with TBW. This may limit the acceptance and adoption of the method by surgeons. Here, we propose a novel technique using TCS, which may be an alternative to the established conventional technique.

The purpose of this study was to evaluate TCS versus TBW for simple transverse patellar fractures in cadaveric and patients' level, aiming to determine whether TCS show superiority over TBW in terms of biomechanical stability in a cadaveric study with benign clinical feasibility and outcomes in patients.

## Materials and methods

### Biomechanical test

With approval of the institutional ethics committee, 15 knee specimens obtained from an aggregate of 31 human cadaver specimens treated with formalin (Department of anatomy, China Medical University) were dissected of soft tissue, leaving quadriceps tendon, patella, and patellar ligament intact. Bone mineral density (BMD) was measured using a dual-energy X-ray absorptiometry scan. BMD was recorded at the previously marked transverse osteotomy line in the centre of each bone. Of 31 scanned specimens, fifteen samples within a close range of BMD were selected. The mean BMD was 1.06 ± 0.292 g/cm^2^. The average cadaver age was 78.3 years (range 62–87), including seven females and eight males. The selected 15 knees were divided into TBW group (group A, 6 cases) or cannulated screws' group (group B, 9 cases). Age, sex, and BMD were distributed homogenously in order to minimize their influence on the results (Table 1). We used an oscillating saw to create a simple transverse patellar fracture model by cutting the patellar horizontally at its midpoint. This was done after measuring the bone mineral density (BMD) of the patellar.

**Table 1:** Distribution of mean age, mean bone mineral density between two groups

Osteosynthesis	Age (years)	Bone mineral density (g/cm^2^)
Three cannulated screws' fixation (*n* = 9)	78.0 ± 5.5	1.06 ± 0.262
TBW (*n* = 6)	77 ± 4.3	1.04 ± 0.197

**Fig. 1:**
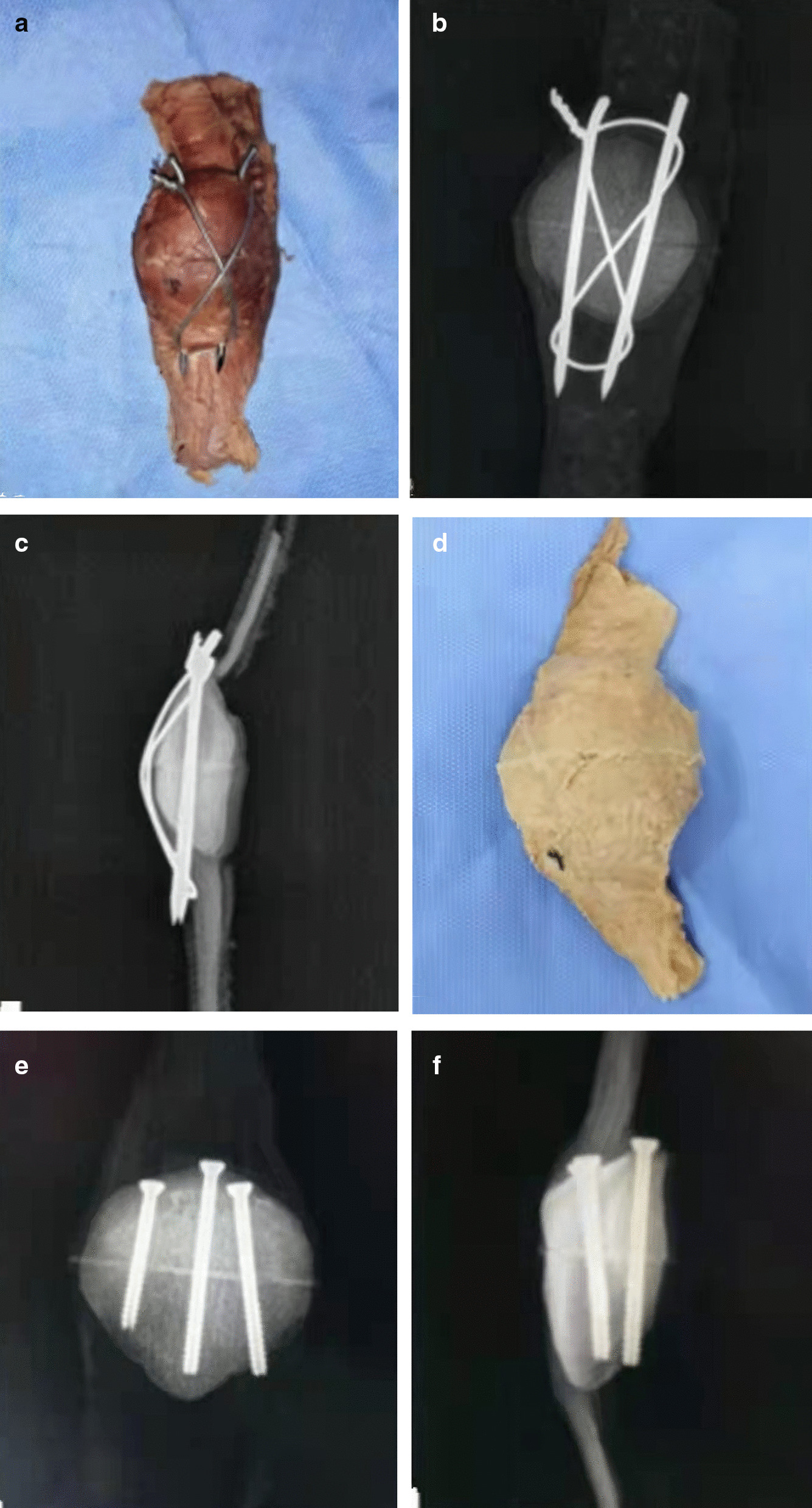
**A** The K-wire tension band technique was performed: **B** anterior–posterior (AP) and **C** lateral radiograph; **D** the three cannulated screws' technique was shown: **E** AP and **F** lateral radiograph

We used two 2.0 mm K-wires to fix the specimens of group A. The K-wires were inserted parallel from the distal to the proximal pole of the patella, crossing the fracture and the quadriceps tendon. We passed a 10-gauge steel wire through the ends of K-wires and made a figure-of-eight with the knot near the proximal part. We twisted the wires to create tension and made sure they had equal turns around each other. Finally, we checked the fracture reduction and the articular surface under direct vision (Fig. 1A–B).

**Fig. 2:**
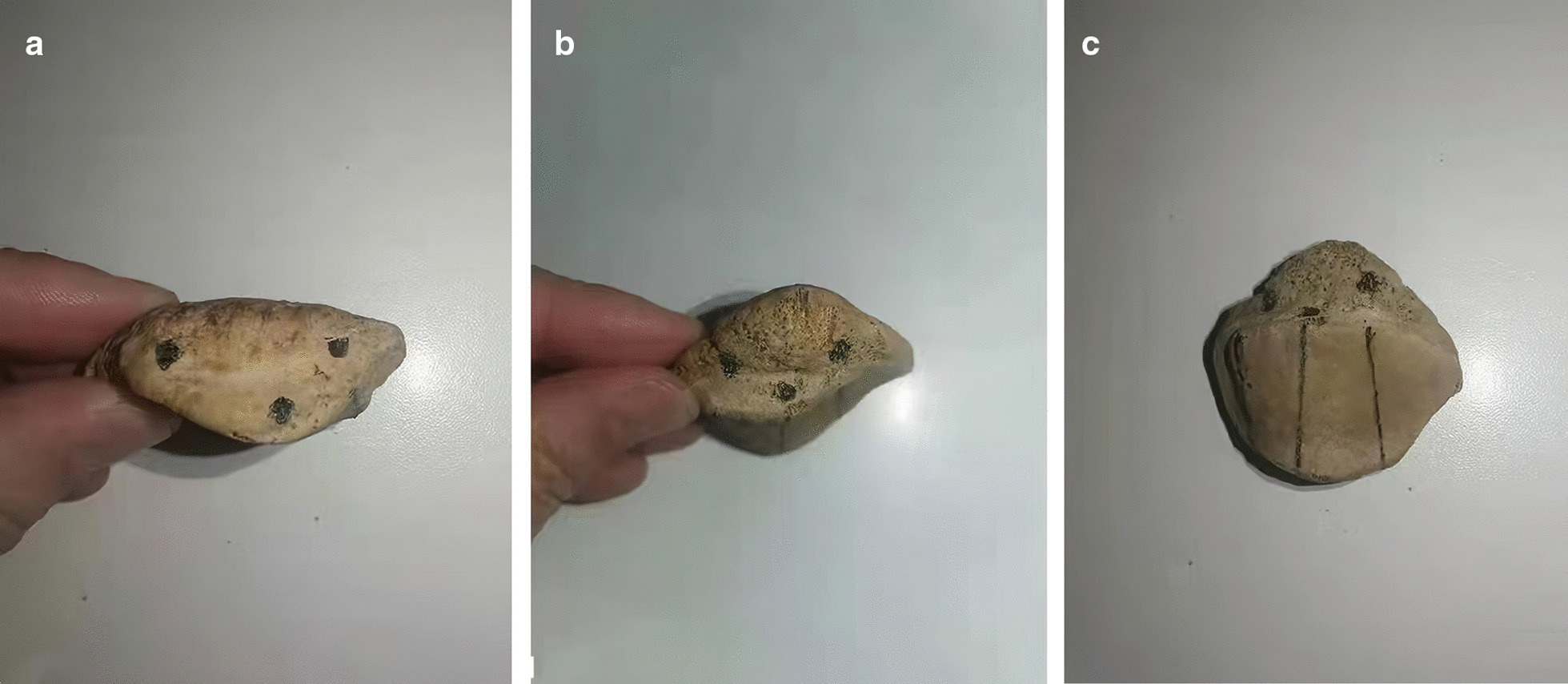
The patella appearance in three cannulated screw group: **A** three entry holes of proximal patella, **B** three exit holes of distal patella; **C** articular surface of patella

In group B, We indicated that we inserted two cannulated screws near the cortical bone (upper) and one near the patellar articular surface (lower) to fix the fracture fragment. The upper guide wires were inserted close to the cortical bone, while the lower guide wire was placed near the articular surface, under direct vision. The guide wires formed an inverted triangle in a 3-dimensional structure (Fig. 2A–C). We drilled along the guide wires and measured the depth before screwing screws to secure the fragment. We checked the reduction of the fragment and the flatness of the articular surface under direct vision (Fig. 1D–F).

**Fig. 3:**
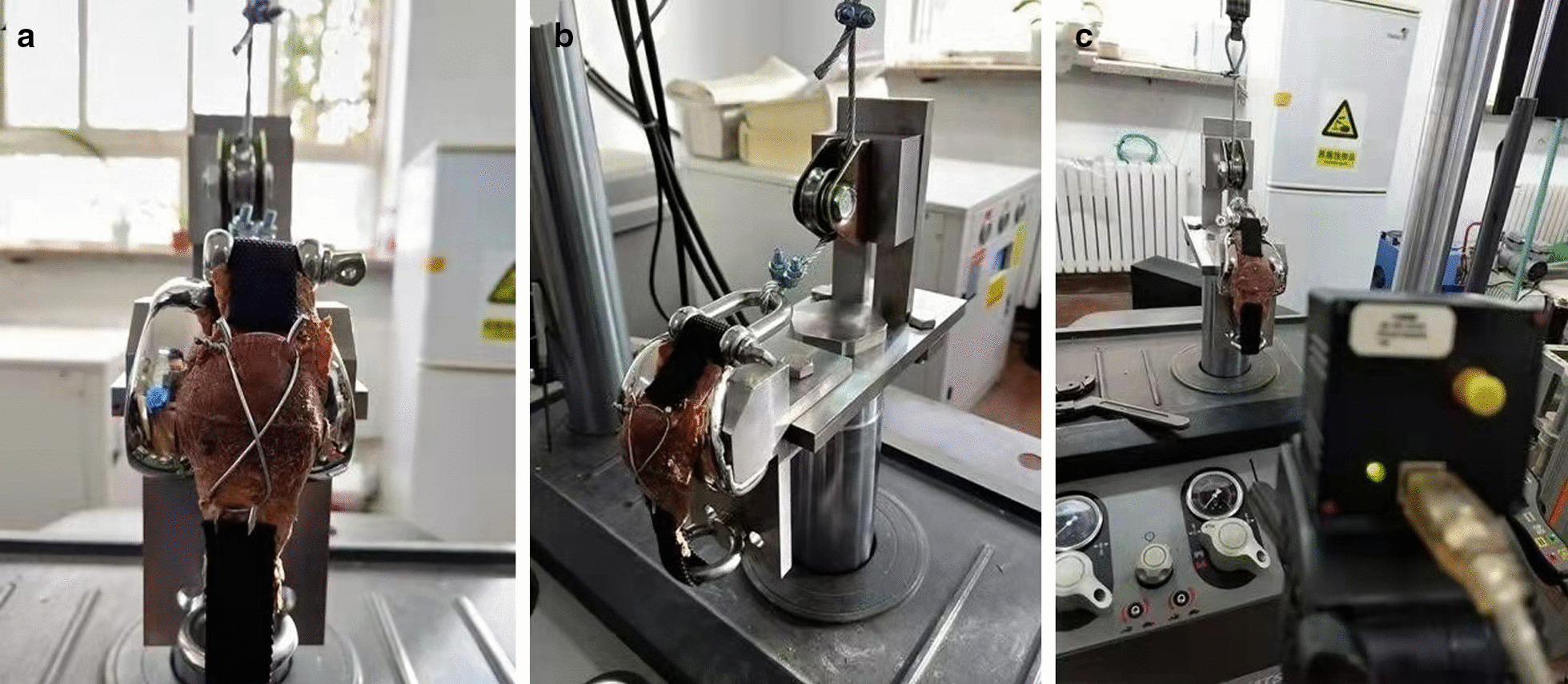
Experiment setup: the quadriceps tendon was attached via a steel cable, which rounded a pulley and was connected to the MTS mechanical and displacement test sensor. **A** Anterior view, **B** lateral view, **C** close view of a high-definition camera continuously measuring the patella separation

All patella were fixed to a customized jig for simulating the natural force direction at 90° knee position, similar to the model described by Schnabel et al. [18]. This angle simulates a situation when standing up from sitting, which has been shown to produce the largest tensile and shear loads on the patella. Specimens were placed on a femoral prosthesis trochlea, with the simple transverse fracture line in the middle of the trochlea. The patellar ligament and quadriceps tendon were tightly connected to a 2.0 mm nylon band and fixed by 2–0 surgical sutures, respectively. The patellar ligament was connected to the lower part of the platform through the nylon band. Loads were introduced to the quadriceps tendon via a steel cable, which rounded a pulley and was connected to the MTS Servo Hydraulic Test System (MTS Corporation, Beijing Branch, China) (Fig. 3).

The patella was subjected to a cyclic load that varied sinusoidally from 25 to 275 N at a frequency of 1/5 Hz. The load started at 150 N and increased to 275 N at the peak of the cycle, then decreased to 25 N at the trough, and returned to 150 N at the end of the cycle. This mimicked the normal force experienced by the patella during walking. A high-definition camera recorded the patella separation at a frequency of 2 Hz. Failure was defined as a fracture displacement exceeding 2 mm, which is clinically regarded as failure.

### Surgical technique

**Fig. 4:**
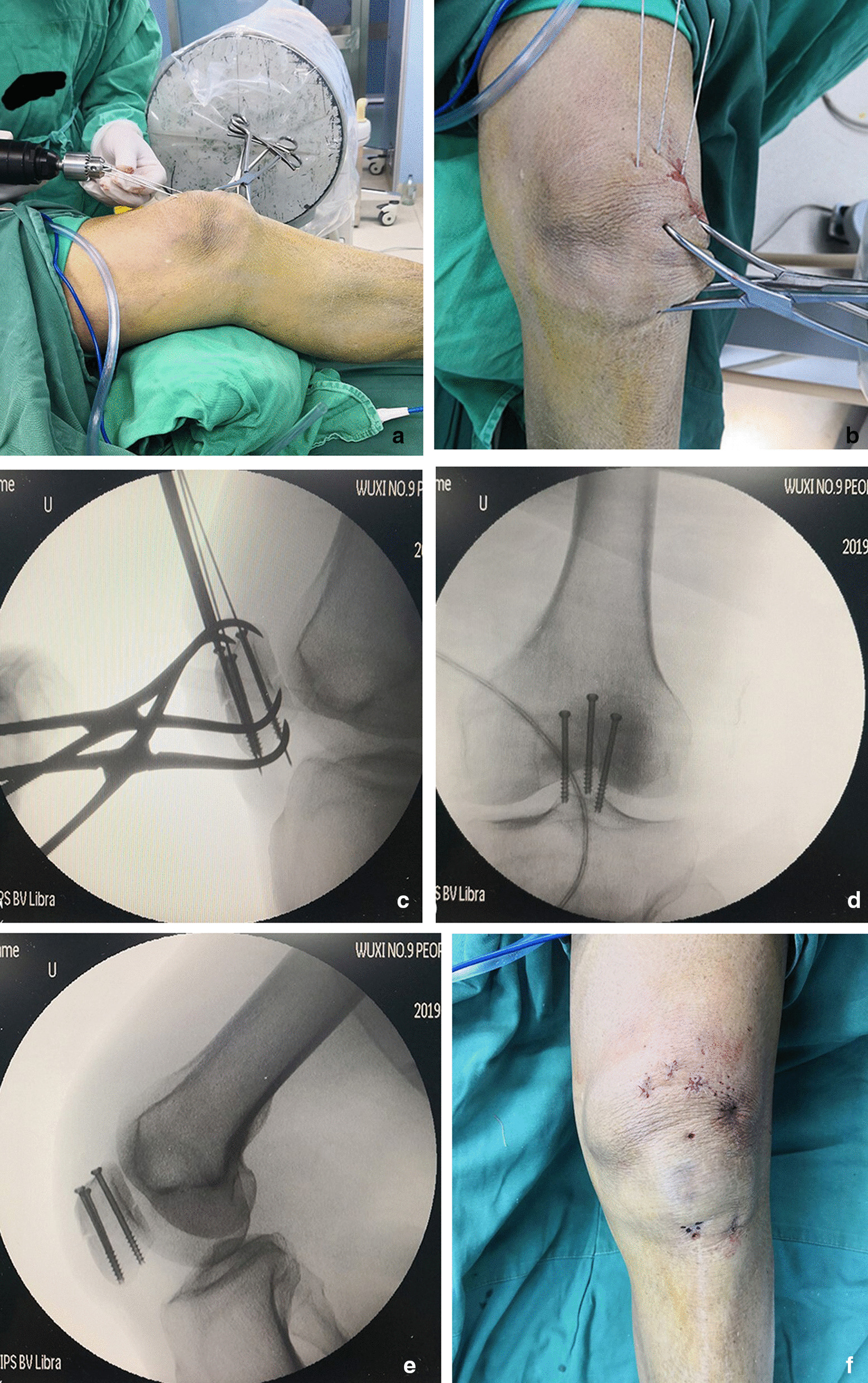
**A**, **B** Fracture was fixed, and three K-wires followed by cannulated screws were inserted under the guidance of C-arm. The images show the alignment of the patella in **C** anterior-lateral (AL), **D** AP, **E** lateral views. **F** The incisions were shown

All patients underwent CT scans to assess the fracture type before surgery. The patient was placed in a supine position on a radiolucent table under anaesthesia during the operation. After standard sterilization, draping, and applying povidone-iodine film, a sterile field was created around the patella. The affected leg was hyperextended as much as possible. The fracture was reduced by aligning the proximal fragment with the distal part. Patella clamps were used to hold the reduction until C-arm radiography was performed. Additional patellar views with 24° external rotation and 34° internal rotation were obtained to optimize the visualization of the lateral or medial facet of the articular surface [12]. The reduction was confirmed before proceeding to fixation. Three K-wires were inserted in an inverted triangle pattern with the knee flexed at 30°. Two K-wires were close to the cortical bone and parallel to the trisection lines on the anteroposterior radiograph of the patella, and the third one was close to the articular surface (Fig. 4A, B). After verifying the length and position of the wires under C-arm radiography, skin incisions were made around the wires. The screw length was measured with a depth gauge before using cannulated drill bits. A 3.5-mm cannulated screw was inserted in the posterior third of the patella, followed by two screws in the middle third [19] (Fig. 4C–E). Following the successful stabilization of the joint, we irritate the articular space with 30 ml of sterile normal saline solution through the superior lateral stab hole. The patellar motion was tested by flexing and extending the knee from 0° to 120° before closing the wound (Fig. 4F).

### Patients

**Fig. 5:**
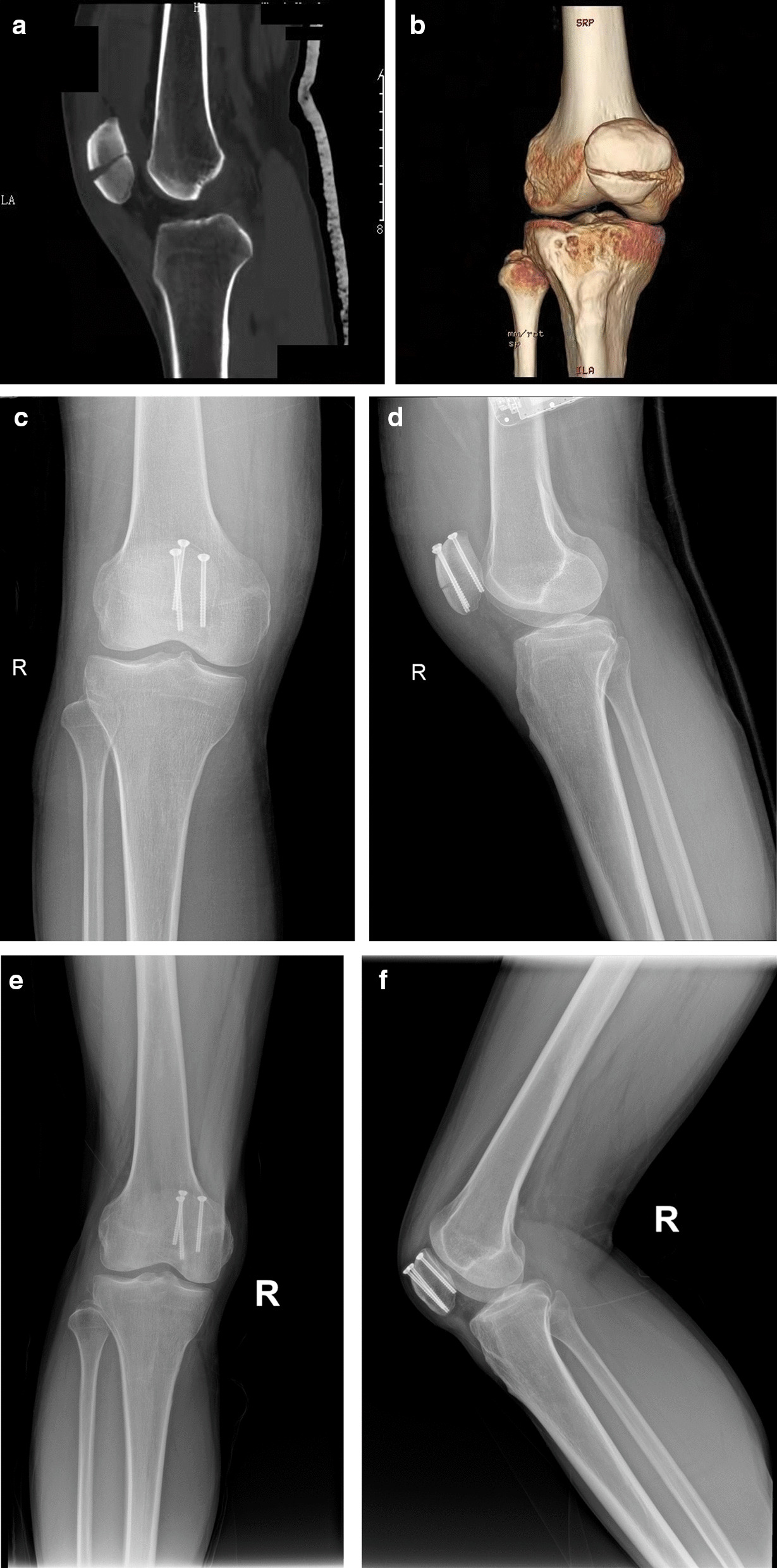
A full series of radiographs showing a typical case's condition before (**A**, **B**), after (**C**, **D**), and during the follow-up period of the surgery (**E**, **F**)

This is a retrospective study of patellar fractures treated TCS by a single surgeon between 2017 and 2021. Institutional review board approval was acquired prior to the initiation of the study. We included 23 cases that met the inclusion criteria, which were: (1) transverse patellar fracture (OTA/AO 34C1.1 type); (2) articular step-off ≥ 3 mm or articular gap ≥ 3 mm without significant step-off [5]; (3) female patients aged ≤ 55 years or male patients aged ≤ 60 years with normal BMD. The patients were 14 men and 9 women, with a mean age of 44.3 years. We performed CT scans and X-rays before surgery to assess the fracture type. An example case is shown below (Fig. 5). We followed up the patients from one month to one year after surgery, or until they were lost to follow-up or had other reasons.

### Clinical evaluation

We used Visual Analogue Scale (VAS) to evaluate pain at one month after surgery, and the Böstman grading scale [1] to measure knee function at six months after surgery. The Böstman grading scale consists of eight items: range of motion, pain, stair climbing, work, atrophy, ambulation assistance, effusion, and giving way. The total score ranges from 30 to 28 points (excellent), 27 to 20 points (good), and < 20 points (unsatisfactory).

### Statistical analysis

Data for fracture displacement were analysed using Wilcoxon rank-sum test and other continuous variants data by independent samples *T*-test after normality and Homogeneity of variance tests. Results are reported as mean ± standard deviation. Statistical significance was accepted at *P* < 0.05. All statistical analyses were performed either using SPSS 25.0 (SPSS Inc., Chicago, IL, USA) or GraphPad Prism 9.0.0 (GraphPad Software, San Diego, California USA).

## Results

**Fig. 6:**
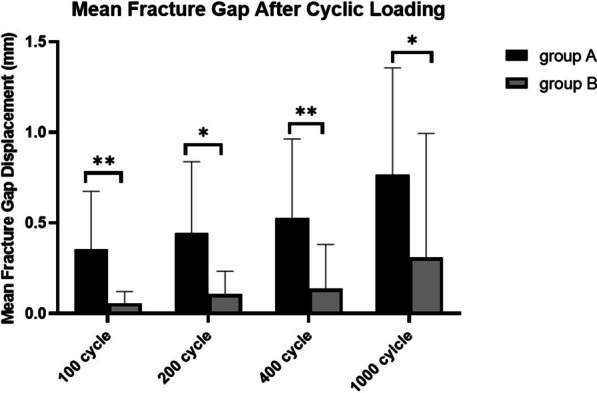
Fracture site displacements of three cannulated screws' fixation (group A) and modified tension band wiring with K-wires (group B) during biomechanical assay

Cannulated screws' fixation group shows credible stability. It was found that the fracture displacement of group B was significantly smaller than that of group A during force loading process. At 100 cycles, the fracture site displacement of group A was 0.36 ± 0.32 mm, and group B was only 0.06 ± 0.06 mm. Group B was much smaller than group A (*P* = 0.0048). After 200 cycles, the fracture site displacement of group A was 0.44 ± 0.40 mm, and group B was 0.11 ± 0.13 mm, which was still noticeably smaller than group A (*P* = 0.0026). After 400 cycles, the fracture site displacement of group A was 0.53 ± 0.44 mm, and group B was 0.14 ± 0.25 mm, which was still significantly smaller than group A (*P* = 0.005). After 1000 cycles, the fracture site displacement of group A was 0.77 ± 0.59 mm, and group B was 0.31 ± 0.67 mm, which showed significantly smaller fracture site displacement (*P* = 0.012) (Fig. 6).

### Clinical experiments

While one patient was lost, We followed up with all patients except one who was lost to follow-up (4.3%). None of the patients had any complications that required metal removal during the follow-up period. The mean operative time was 44.8 ± 8.4 min, the mean VAS score was 1.96 ± 0.75 one month after surgery, and the mean Böstman grading scale 6 month after surgery was 28.7.

## Discussion

Tension band construct developed in the 1950s by the Arbeitsgemeinschaft für Osteosynthesefragen / Association for the Study of Internal Fixation (AO/ASIF) for transverse fractures is ideal because it uses the compression of the braces during bending movements, which allows early mobilization [20]. This technique converts the anterior tension forces produced by the extensor mechanism and knee flexion into compression forces at the articular surface. The technique has been the mainstream for the fixation of the transverse patellar fracture [21, 22]. It provides stable fixation, nevertheless it is not without drawbacks, such as hardware discomfort or complications such as irritation or infection [5, 23–25]. Although the rates of these downsides may be largely reduced by replacing the metal wires with nonabsorbable suture wire (FiberWire/Ethibond) [26, 27], removal of troublesome material is still needed in up to 60% of patients [5–7].

A meta-analysis involving 949 patients by Zhang et al. [28] compared 581 patients with Kirschner wire tension bands and 368 patients with alternative fixation methods (cannulated screws, cable pin, and ring pin). Their pooled analysis showed alternative fixation methods had lower complication rates and better outcomes considering flexion range of motion, pain scores (VAS), and knee function scores (Böstman joint function score, Iowa knee score, and Lysholm score). They attributed these impressive results to minimal invasion and better biomechanical property that closed reduction and fracture fixation using cannulated screws' system or others had.

Previous reports have shown numerous advantages of the percutaneous fixation technology, including the avoidance of extended incision and preservation of the blood supply to the patella [29–33]. Besides, the low profile of the cannulated screw with less protuberance may decrease the irritation to the soft tissue. Finally, postoperative pain control is more accessible because extended incisions are prevented because extended incisions are prevented.

Cannulated screws with tension band technique had been tried to treat transverse patellar fractures. However, rare studies proved the biomechanical stableness of the technique merely using cannulated screws in treating patella fractures since it was first proposed in 1993 by Appel et al. [29]. The technique avoids extensive soft tissue dissection to preserve blood supply and viability of skin flaps, which may lead to far less disability and discomfort caused by post-surgery pain, substantially shorter hospitalization, and accelerated recovery. In contrast, tension band construct requires a substantial dissection with a long skin incision to provide visualization of the fracture surface as well as the joint surface. This gives rise to a substantial amount of adhesion and prolonged disability, as well as a cosmetically unpleasing scar.

In our study, three cannulated screws' fixation for simple transverse patellar fracture showed better stability than traditional K-wire tension band technique. Compared to other treatments, here we showed that employing cannulated screws merely performs a promising application in the simple type of patellar fracture (AO/OTA 34C1.1) in aspects of success rate, the incidence of complications and infection, and post-operative pain.

There are several limitations of this study. First, the maximum loads of the two fixations were not measured. Second, we were only able to simulate the situation of a simple transverse fracture as most other cadaveric studies do. This scenario is uncommon in clinical practice, which limits the generalizability of our findings to more complex cases. Third, we used specimens that were preserved with formalin solution, which may alter the mechanical properties of the patella and soft tissue.

Therefore, we think it is essential to follow the indications for this technique to obtain satisfactory outcomes. The cannulated screws' technique is suitable for transverse patellar fractures in the middle third of the patella (AO/OTA 34C1.1) without significant displacement and comminution of the fragments. These fractures usually involve disruption of the extensor mechanism that requires open surgery and repair. Benjamin et al. [22] also recommend that screw fixation be used in patients with adequate bone quality. Moreover, based on the AO principle of anatomical reduction and compression, the screws must be securely anchored in the bone, but this may not be possible in cases of comminution and/or osteoporosis.

As a closed reduction technique, the main difficulty in the surgical process is that the surgeon cannot directly see or feel the articular surface and therefore requires more training and experience. Computed tomography before surgery is preferable, as recent studies have shown that radiographs often underestimate the complexity of many patellar fractures [23, 24].

In conclusion, percutaneous cannulated screws for simple transverse patella fractures are a promising option with minimal incision, less soft tissue damage, and possibly lower removal rate of bothersome material.

## Data Availability

The datasets used and/or analysed during the current study are available from the corresponding author on reasonable request.

## Abbreviation

TBW: Tension band wiring
CRPF: Closed reduction and percutaneous fixation
TCS: Closed reduction and percutaneous fixation with three cannulated screws
RCT: Randomized controlled trial
ORIF: Open reduction and internal fixation
BMD: Bone mineral density
VAS: Visual Analogue Scale
AO/ASIF: Arbeitsgemeinschaft für Osteosynthesefragen/Association for the Study of Internal Fixation
AP: Anterior–posterior
AL: Anterior-latera
